# Microvascular Fluid Exchange: Implications of the Revised Starling Model for Resuscitation of Dengue Shock Syndrome

**DOI:** 10.3389/fmed.2020.601520

**Published:** 2020-12-22

**Authors:** Dinh The Trung, Huynh Trung Trieu, Bridget A. Wills

**Affiliations:** ^1^Oxford University Clinical Research Unit, Ho Chi Minh City, Vietnam; ^2^Paediatric Intensive Care Unit, Hospital for Tropical Diseases, Ho Chi Minh City, Vietnam; ^3^Centre for Tropical Medicine and Global Health, Nuffield Department of Medicine, University of Oxford, Oxford, United Kingdom

**Keywords:** revised Starling model, dengue shock syndrome, fluid management, endothelial glycocalyx layer, tight junction, colloid, crystalloid

## Abstract

Dengue is the most common mosquito-borne viral infection in the world. The most feared complication is a poorly understood vasculopathy that occurs in only a small minority of symptomatic individuals, especially children and young adults, but can result in potentially fatal dengue shock syndrome (DSS). Based mainly on expert opinion, WHO management guidelines for DSS recommend prompt infusion of a crystalloid fluid bolus followed by a tapering crystalloid fluid regimen, supplemented if necessary by boluses of synthetic colloid solutions. However, following publication of a number of major trials undertaken in other, primarily adult, critical care scenarios, use of both synthetic colloid solutions and of fluid boluses for volume expansion have become controversial. Synthetic colloids tend to be used for severe DSS cases in order to boost intravascular oncotic pressure, based on the classic Starling hypothesis in which opposing hydrostatic and oncotic forces determine fluid flow across the microvascular barrier. However, the revised Starling model emphasizes the critical contribution of the endothelial glycocalyx layer (EGL), indicating that it is the effective oncotic pressure gradient across the EGL not endothelial cells *per se* that opposes filtration. Based on several novel concepts that are integral to the revised Starling model, we review the clinical features of DSS and discuss a number of implications that are relevant for fluid management. We also highlight the need for context-specific clinical trials that address crucially important questions around the management of DSS.

Dengue is increasingly being recognized as a major threat to global health, especially among people living in endemic areas who are exposed to multiple infections with different serotypes. Although the majority of symptomatic infections manifest as a self-limited illness characterized by fever and systemic symptoms, life-threatening complications can develop. The most feared complication, particularly prevalent in children and young adults experiencing secondary infections, is a poorly understood vasculopathy that presents as a systemic vascular leak syndrome (1). The vasculopathy reverses spontaneously after about 48 h, but plasma losses can be substantial during this time, potentially resulting in dengue shock syndrome (DSS) (1). Plasma leakage is typically accompanied by thrombocytopenia and hemostatic derangements that favor bleeding (2, 3). Minor bleeding is often observed when tissue integrity is breached (e.g., during venesection) but in individuals with prolonged or profound shock, in whom the additional effects of tissue hypoxia and acidosis compound the basic hemostatic derangements (3), the risk for major mucosal bleeding increases. Such bleeding is often from the gastrointestinal tract and can be difficult to detect; thus in DSS cases who fail to respond to fluid resuscitation, the possibility of occult bleeding should always be considered (1).

Severe leakage resulting in DSS is seen less frequently in adults than children (4, 5), likely reflecting the higher intrinsic permeability of pediatric microvessels (6), as well as immaturity of the homeostatic mechanisms that help to prevent cardiovascular decompensation in the face of volume depletion (5). However, in settings where the local epidemiology favors symptomatic dengue in later life the elderly are also at greater risk for severe dengue, probably due to the complex interplay of factors such as vascular aging, immune senescence, and the presence of chronic diseases and comorbidities (7).

In this review we highlight some of the current controversies relating to DSS fluid resuscitation and consider how evolving knowledge of the physiological principles governing fluid dynamics at the microvascular level can help us to understand the clinical features observed in DSS and potentially be of use in guiding fluid management of shock.

## Controversies Around Fluid Resuscitation of Dengue Shock Syndrome

In the 1960s, when DSS was first described mortality rates were high, but recognition of the marked intravascular volume depletion and the urgent need for fluid resuscitation led to a notable improvement in outcomes. Great care is needed however to limit iatrogenic complications, in particular development of respiratory compromise due to fluid overload, especially in settings where HDU/ICU facilities with the capacity to provide advanced respiratory support are scarce. The World Health Organization (WHO) dengue management guidelines recommend prompt infusion of 10–20 ml/kg crystalloid fluid boluses for children with established DSS, followed by a tapering crystalloid fluid regimen supplemented by boluses of synthetic colloid solutions as necessary (1). Management strategies for high-risk groups require adjustment in line with physiological norms [for example infants have much higher basic fluid requirements than older children (8)] or to balance competing risks in those with comorbidities [for example elderly individuals with cardiac and renal conditions (7)]. However, in centers of excellence outcomes for DSS are generally good, although WHO estimates indicate case fatality ratios can be up to 2.5% (1).

The evidence for the current WHO management guidelines is limited and the recommendations are based primarily on expert opinion and some observational data (8, 9), although several relatively small randomized controlled trials support the general principles (10–13). Over the last 15 years however, following publication of a number of major trials in unselected adult ICU populations, use of synthetic colloid solutions for volume expansion has become highly controversial in western settings (14–18). When isotonic crystalloids alone are deemed inadequate for resuscitation international guidelines now typically recommend albumin solutions (19), although the evidence base for this guidance is also limited (20, 21). However, in the LMIC settings where DSS commonly occurs albumin is prohibitively expensive and synthetic colloids, particularly hydroxyethyl starch solutions, continue to be widely deployed as rescue therapy in the belief that this strategy reduces the risk of fluid overload and minimizes the need for respiratory support (10, 22). Typically, more than a third of pediatric DSS cases receive a synthetic colloid as part of their fluid resuscitation regimen.

Another controversial issue is the use of fluid boluses for resuscitation. Rapid correction of profound hypovolemia has always been accepted as an important feature of shock resuscitation, but there is very little formal evidence available in this regard. A large trial in African children (FEAST) with impaired perfusion associated with severe febrile illness of mixed etiology yielded unexpected but unambiguous results, demonstrating that fluid boluses were associated with significantly increased 48-h mortality (23). However, a WHO expert review committee concluded that few of the study participants had overt shock and WHO's revised Emergency Triage and Treatment (ETAT) guidelines continue to advise use of fluid boluses for children with clear evidence of shock, albeit with the recommendation to use smaller volumes of fluid over longer periods of time (24, 25). Although subsequent reanalysis of the trial data indicated that the increased mortality risk was apparent across all definitions of shock (26), a recent review of available guidelines indicates that bolus therapy remains universally recommended for children with established shock (27).

Finally, although blood transfusion is considered life-saving for individuals with DSS and severe bleeding, identification of occult bleeding can be difficult. WHO management guidelines recommend prompt administration of fresh blood as soon as significant bleeding is suspected (1), but evidence to support particular regimens—how much of which blood product, over what time interval, with what particular therapeutic goal—is lacking.

## Dengue Associated Vascular Leakage in the Context of the Revised Starling Model

In health, intravascular volume is regulated within tightly circumscribed limits by complex homeostatic mechanisms. Historically, the general principles governing fluid resuscitation developed in parallel with evolving knowledge of human physiology and vascular biology. Starling originally hypothesized in 1896 that opposing hydrostatic and oncotic pressures in the capillaries of the microcirculation determined fluid flow between the intravascular and interstitial fluid compartments (28). In line with this model administration of colloids became an accepted strategy for management of hypovolaemic shock during the latter half of the 20th century, aiming to boost intravascular oncotic pressure and favor fluid re-absorption (29). However, although considerable experimental evidence accrued during this period to confirm the general validity of Starling's basic principle, research to better define the physiological and structural characteristics of the microvascular barrier also established the critical contribution of a hitherto unrecognized entity, the endothelial glycocalyx layer (EGL), leading to a more nuanced interpretation of the classic model, now generally referred to as the revised Starling model (30, 31).

The revised Starling model emphasizes the crucial role of the EGL in fluid filtration across the “surface glycocalyx endothelial complex.” Although the detailed structure remains to be elucidated, we now know that the EGL consists of an ordered matrix of glycoproteins and proteoglycans anchored in endothelial cells and extending throughout all vascular beds. Plasma proteins such as albumin adhere to and consolidate the structure, resulting in formation of a negatively-charged size-selective barrier that is semipermeable to macromolecules (32, 33). Cellular elements of blood are excluded from the layer, but different macromolecules are able to penetrate and gain access to the underlying cellular filtration mechanisms depending on their size, charge, and shape (34). Thus, colloid molecules are typically retained in the free-flowing plasma compartment for a period of time, evidenced by hematocrit dilution effects, before distributing more widely across the body fluid compartments (35, 36). Although the EGL is very difficult to visualize there is evidence to indicate that it is compromised in many inflammatory and infectious conditions, with increases in plasma levels of certain biomarkers (e.g., heparan sulfate, syndecan-1) considered to be indicators of glycocalyx injury (32, 33).

**Figure 1:**
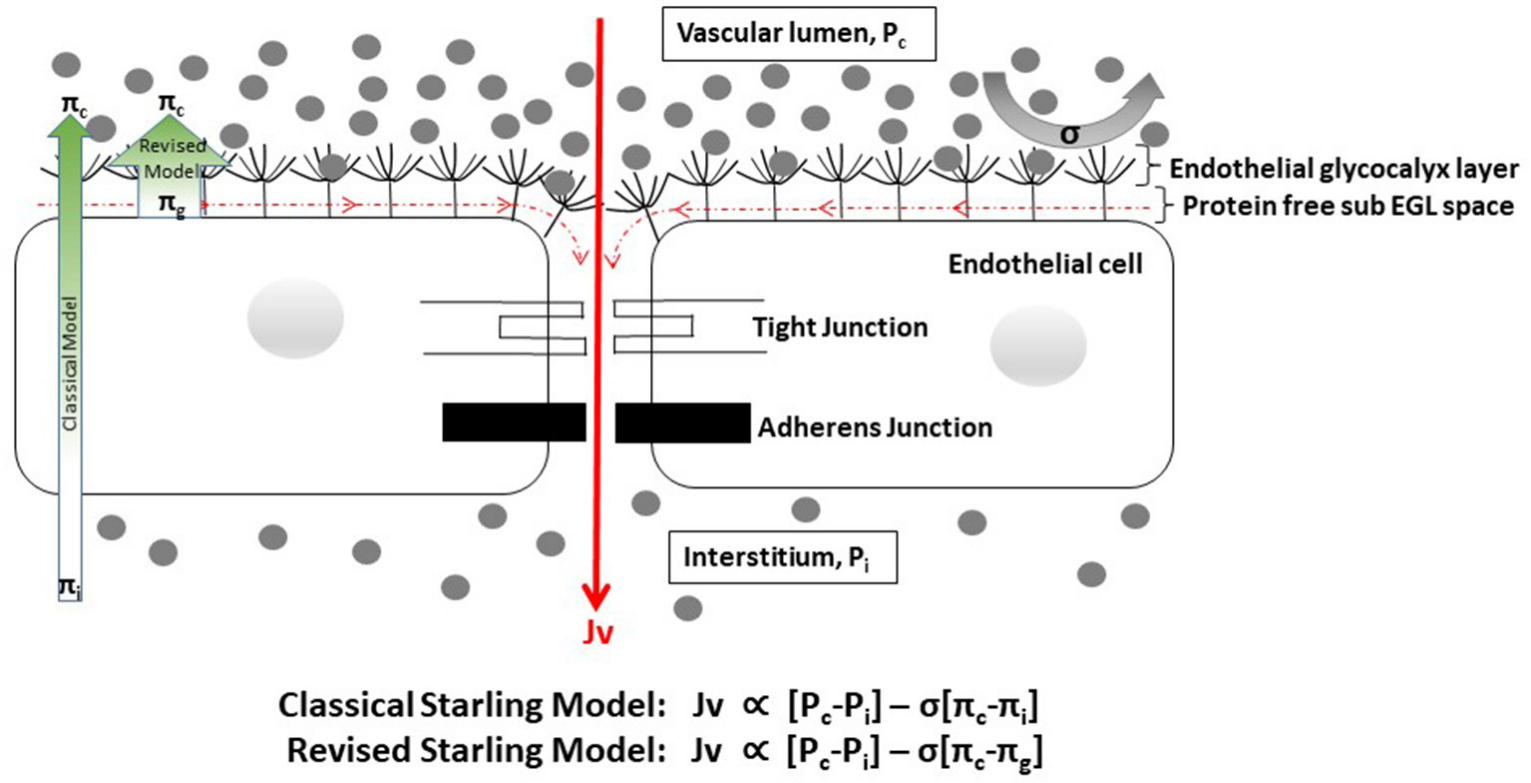
Cartoon depicting factors influencing fluid flow through the endothelial barrier [adapted from (37)]. EGL, endothelial glycocalyx layer. Gray dots represent protein molecules. Fluid flow (Jv) is proportional to the hydrostatic gradient (i.e., the difference between the capillary {*P*_*c*_} and interstitial {*P*_*i*_} hydrostatic pressures) minus the product of the reflection coefficient {σ} and the oncotic pressure gradient [i.e., the difference between the capillary (π_*c*_) and interstitial or sub-glycocalyx {π_*i*_ or π_*g*_} oncotic pressures]. The reflection coefficient indicates the ease with which molecules either penetrate the EGL or are deflected back into free-flowing plasma, and varies between 0 and 1. In health σ is close to 1 for albumin and most other plasma proteins. In dengue σ is likely to be reduced, reflecting changes to the EGL structure. The EGL forms the primary barrier to movement of fluid and plasma contents from the intravascular to the interstitial spaces. Fluid that enters the sub-glycocalyx space flows toward the inter-endothelial clefts and is channeled at high velocity to the interstitium, due to the physical characteristics of the cleft architecture and the narrow width of the gaps in the structures that bind the cells together. This high velocity fluid movement prevents back diffusion of interstitial proteins toward the protected sub-glycocalyx space, which remains effectively protein free. Fluid and proteins that get to the interstitial space are reabsorbed back into the intravascular space via the lymphatic system. In line with the revised Starling model the oncotic pressure gradient from the protein free sub-glycocalyx space to plasma (broad green arrow) is greater than expected with classical Starling model (narrow green arrow), and fluid losses to the interstitium are much lower than previously thought.

**Figure 2:**
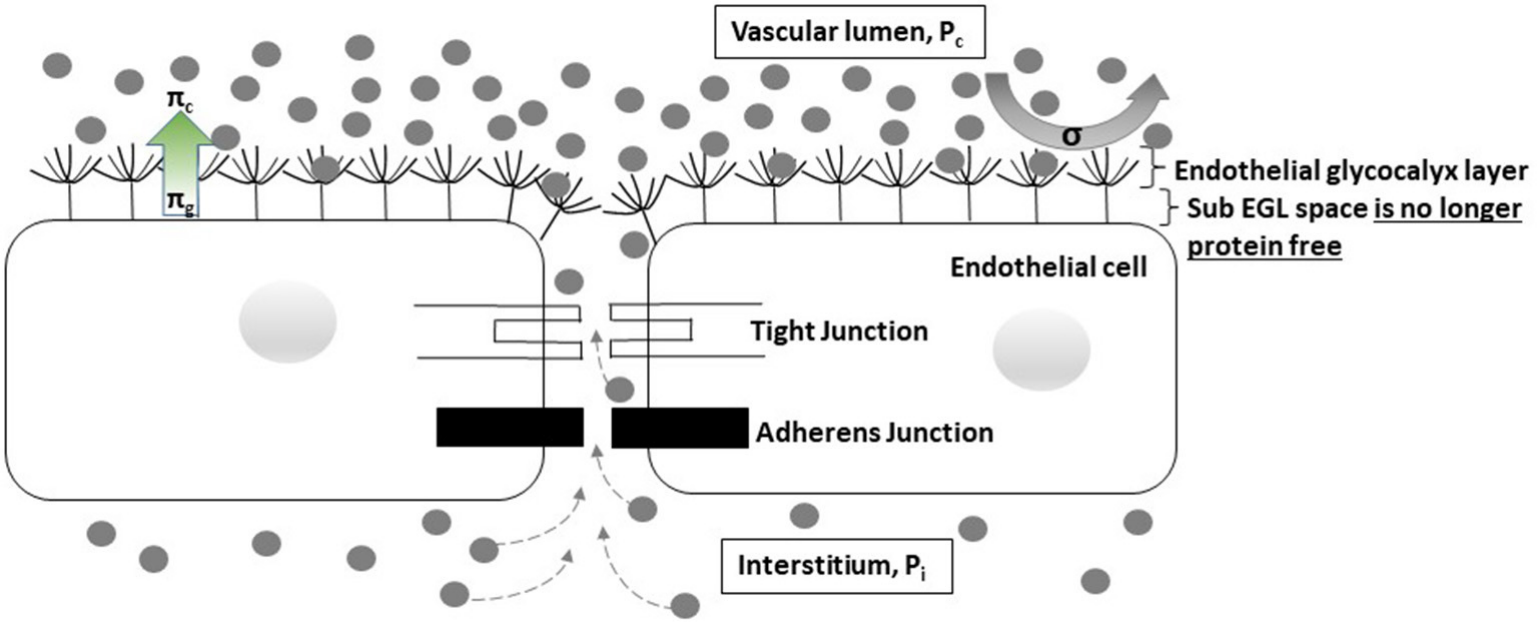
Cartoon depicting the expected changes associated with profound shock. In profound shock the hydrostatic pressure gradient from the vascular lumen to the interstitial space is abolished (*P*_*c*_ = *P*_*i*_). Fluid and proteins are able to diffuse back through the inter-endothelial cleft to the sub-glycocalyx space, allowing reabsorption of about 500 ml (autotransfusion) to occur. The capillary pressure rises and a new hydrostatic pressure gradient is established within about 30 min. Fluid filtration restarts but at a low level. However, the oncotic pressure gradient opposing filtration is also reduced since some protein is now present in the sub-glycocalyx protected space. Following successful resuscitation, hydrostatic pressure improves, intercellular cleft fluid flow increases, and protein is washed out from the sub-glycocalyx space until both *P*_*c*_ and π_g_ are restored to normal.

Several important consequences of the revised Starling model need to be acknowledged. Originally, plasma was thought to be filtered to the interstitial space at the arteriolar end of capillaries under a dominant hydrostatic pressure gradient, with the majority of the fluid later being re-absorbed at the venular end under a dominant oncotic pressure gradient. Now however, it is clear that the effective oncotic pressure gradient lies within microvessels, i.e., it is the local colloid osmotic pressure difference between free-flowing plasma and the subglycocalyx space that opposes filtration. As the subglycocalyx space is virtually protein free in comparison with interstitial fluid protein levels, the oncotic pressure gradient opposing fluid filtration is greater than expected with the original model, and thus the rate of fluid loss to the interstitial space is much lower than previously thought (Figure 1) (31, 35). Secondly, once in the protected subglycocalyx space fluid flows toward the inter-endothelial clefts and is directed at high velocity, determined by the physical characteristics of the clefts, the junctional strand, and the tight and adherens junctions that bind adjacent endothelial cells together, into the interstitial space. This high-velocity stream is effectively one-way, ruling out the possibility of steady state fluid reabsorption as dictated by the original Starling model (31, 35–37). Any fluid that is filtered returns to the circulation via lymphatic channels; in response to increased pressure in local lymphatics, fluid flow toward the thoracic duct increases markedly, but the protein (primarily albumin) content of the returned fluid is low compared to plasma (31). There is however, a situation in which interstitial fluid can flow back up the inter-endothelial clefts and enter the subglycocalyx space. If capillary pressure is severely reduced (e.g., in profound shock) the pressure dynamics within the clefts alter and there is an immediate but transient reversal from filtration to reabsorption (so called autotransfusion) until the capillary pressure rises again and a new steady state condition with reduced filtration becomes established after about 30 min (Figure 2) (35–37).

Considering the observed clinical features of DSS, a slow sustained plasma leak evolving over several days is consistent with the revised Starling model. Characteristically shock occurs late in the illness episode (day 4–6), heralded by increasing hemoconcentration that is followed by a rise in diastolic pressure with concomitant narrowing of the pulse pressure (PP). Secondly plasma albumin concentrations are often markedly reduced (<20 gm/L) immediately after initial volume replacement counterbalances the effect of hemoconcentration, but rapidly return to normal within 24–48 h as the vasculopathy resolves—i.e., within a timeframe consistent with redistribution rather than consumption and *de novo* synthesis. The revised Starling model suggests that a slow plasma leak would be balanced by a proportionate increase in lymphatic return that, together with cardiovascular, renal, and adrenal compensatory mechanisms, should initially serve to ensure that plasma volume remains close to normal. However, over time progressively worsening hypo-albuminaemia would reduce the colloid osmotic gradient between free-flowing plasma and the subglycocalyx space, thereby increasing filtration into the interstitial space to a level that outstrips the capacity of the lymphatic system to return the filtered fluid to the circulation. Eventually plasma volume becomes compromised, but by the time cardiovascular decompensation actually occurs the intrinsic homeostatic mechanisms are maximally upregulated, explaining the counterintuitive rise in diastolic pressure that is observed.

There is evidence to suggest that dengue-associated vascular leakage may result from disruption of the EGL itself. In secondary dengue infections viral replication is amplified, resulting in a range of downstream effects that perturb microvascular function without obviously altering the architecture of the endothelial cell layer. As yet details of the process by which this occurs remain obscure, in part due to difficulties in visualizing the structure of the EGL *in vivo*, but increased levels of several glycocalyx constituents have been documented, with some evidence indicating associations with the timing and severity of leakage (38–40), suggesting that structural characteristics of the EGL are altered during human DENV infections. In addition imaging studies of the sublingual microcirculation have shown an increase in the perfused boundary region within microvessels consistent with thinning of the EGL (41). *In vitro* and mouse model studies have demonstrated that dengue nonstructural protein 1 (NS1) can disrupt the surface glycocalyx both directly and/or through inflammatory-dependent pathways (42, 43). A number of mediators released from infected or activated cells (chymase, tryptase, angiopoetin-2, platelet activating factor, and various cytokines) have been shown to have *in vitro* effects on the integrity of inter-endothelial tight junctions (44–46), with increased levels of some of these mediators also shown to correlate with disease severity in DENV infected individuals (44, 46–48), while levels of mediators that help to maintain endothelial integrity (angiopoietin-1, sphingosine1-phosphate) were reduced (47, 49). In one study plasma concentrations of claudin-5, an integral component of inter-endothelial tight junctions, were increased and the levels were strongly associated with the severity of vascular leakage (39). One hypothesis is that during acute dengue the function of the EGL, the primary vascular barrier, is compromised to some degree in all individuals (potentially via NS1 related effects), resulting in some increase in vascular permeability. If other downstream structural components of the barrier, such as the tight junctions within the inter-endothelial clefts, are also disrupted (potentially via other mechanisms as indicated above), then major increases in permeability occur increasing the risk for DSS.

These revised concepts of vascular physiology have important implications for management of DSS. Conventionally a PP < 20 mmHg associated with signs of impaired perfusion defines the threshold for diagnosis of established DSS (1), although patients may still appear deceptively well. By this time however marked hemoconcentration is often present, with hematocrits in excess of 50–55% not infrequently documented in children presenting with DSS (from a likely baseline of 35–40%), suggesting contraction of intravascular volume by 40–50% in severe cases (11). If fluid replacement is not commenced promptly the systolic pressure falls rapidly and irreversible shock and death may follow despite aggressive resuscitation (9, 22). Although crystalloids have a better safety profile, given the anticipated differential distribution of infused crystalloid/colloid fluids between body fluid compartments, many clinicians have hitherto opted to use a colloid immediately for children with profound shock (1, 10, 50). There is also some evidence suggesting that colloid molecules may transiently interact with a dysfunctional EGL structure helping to restore barrier function (37, 51). However, considering that (a) when capillary pressure is close to zero there is little difference in the disposition of crystalloids vs. colloids until hydrostatic pressure is restored to a level at which filtration recommences, (b) crystalloid resuscitation may be preferable to repay the autotransfusion and re-establish the subglycocalyx protected space (35, 37), and (c) that dengue-associated disruption of the EGL is very likely to impact its functional characteristics, there is a good rationale for selecting a crystalloid even in this circumstance. Similarly, there is a scientific rationale for avoiding use of large fluid boluses during profound shock, since the effect may be to boost capillary pressure to a point at which hyperfiltration occurs transiently, in line with the findings of the FEAST trial (23). In addition, in patients with septic shock plasma heparan sulfate levels both predicted mortality and were significantly associated with the volume of intravenous fluids administered independent of disease severity, suggesting that there may be a relationship between infused fluid volume and ongoing EGL damage (52). A summary of the possible advantages and disadvantages of crystalloid and colloid fluid administration for DSS resuscitation is presented in Table 1, considered in the light of the revised Starling model.

**Table 1:** Effects of administering the same volume of crystalloid and colloid fluids for DSS volume resusication, considered in the light of the revised Starling model.

**Characteristic**	**Isotonic Crystalloid Solutions**	**Human-derived Colloid Solutions (albumin, fresh frozen plasma)**	**Synthetic Colloid Solutions (dextran, gelatin, or starch based)**
Volume of distribution	Rapid distribution throughout the extracellular fluid {ECF} space, including the intravascular space (free flowing plasma plus the endothelial glyocalyx layer {EGL}) and the interstitial space.	Large molecules penetrate the EGL slowly, depending on their size, charge and shape. Thus colloids distribute within free flowing plasma initially, and can exert an oncotic effect to draw water from the EGL (estimated to be about 700 ml) back into free flowing plasma. Gradually the molecules distribute throughout the ECF.	
	Small hematocrit dilution effects.	Greater initial hematocrit dilution effect than with crystalloids, reflecting in part the movement of water from the EGL back into plasma.	
Influence on plasma oncotic pressure	Plasma oncotic pressure falls. Immediate volume expansion is less than with colloids, but since reabsorption of interstitial fluid does not occur from microvessels, the difference is less than expected with the classic Starling model.	Plasma oncotic pressure rises, and water is drawn in from the EGL to increase free flowing plasma volume by more than the volume of fluid infused. However, the increased oncotic pressure does not result in reabsorption of interstitial fluid from microvessels, as expected with the classic Starling model.	
Influence on capillary hydrostatic pressure	Rising capillary pressure results in increased fluid filtration.	Capillary pressure rises but, counter-balanced by the concomitant increase in plasma oncotic pressure, the increase in fluid filtration is less marked than with crystalloids.	
	Once the capacity of the lymphatic system to reabsorb flitered fluid is exceeded, any excess remains in the interstitial space potentially contributing to fluid overload.	Once the capacity of the lymphatic system to reabsorb flitered fluid is exceeded, any excess remains in the interstitial space potentially contributing to fluid overload.	
Effects in profound shock	If hydrostatic pressure is very low or absent, high-velocity fluid flow through the inter-endothelial clefts toward the interstitial space ceases, and water and solutes can diffuse back toward the protected subglycocalyx space, allowing some reabsorption to occur until capillary pressure rises again and a new steady state condition is established. No difference is expected effects between crystalloid and colloid fluids in these circumstances.		
Potential effects when EGL barrier function is itself compromised	Not known	If the barrier function of the EGL is disrupted colloid molecules may penetrate the layer more easily, reducing the effects on plasma oncotic pressure that allow EGL water to be absorbed back into free flowing plasma.	
Potential to restore structural/functional characteristics of EGL	No	Both albumin and FFP have additional effects on intravascular volume dynamics that are independent of their effects on plasma oncotic pressure. The underlying mechanisms remain unclear but may be related to an interaction with the EGL, that in some way restores the barrier function of the layer when damaged.	Some evidence also suggests that certain synthetic colloids may protect or restore the EGL layer through an unknown mechanism, though with a less marked effect than human derived colloids.
**Other important adverse effects associated with use of these fluid groups for resuscitation**			
Electrolyte disorders	Well-recognized, depending on the specific solution used	Low risk	Low risk
Acid base disturbances	Large volumes of certain crystalloids (e.g., 0.9% saline) can cause metabolic acidosis and hyperchloremia	Very low risk with large volumes	Low risk with large volumes of dextran/starch solutions. Gelatin solutions contain little chloride.
Coagulation disorders/bleeding risk	Dilutional effects on coagulation tests, but no apparent increase in bleeding risk	Dilutional effects on coagulation tests, but no apparent increase in bleeding risk	Both dilutional effects and distinct product specific effects occur, that do increase bleeding risk
Acute kidney injury	Very low risk	Low risk	Definite risk, varies with product
Allergic reactions	Very low risk	Albumin—low risk	Definite risk, varies with product
		FFP—higher risk	

Existing evidence supports the use of crystalloids for resuscitation of children with compensated DSS but as yet there are no comparative data for profound shock. Similarly, there are no data for DSS cases that fail to respond to initial resuscitation with a crystalloid or who subsequently decompensate after a period of stability. Yet these are the circumstances when clinicians worry most. The findings of the major crystalloid/colloid comparison studies carried out in mixed adult ICU populations across Australia, New Zealand, and Europe are clear (17, 18, 53), but DSS is very rarely seen in these settings and the dilemmas facing clinicians working in environments with completely different disease and patient profiles must not be ignored (54). It is also important to recognize that there are a number of reasons why the safety profile of synthetic colloids in children with DSS may differ from that observed in unselected adult ICU populations: most children with DSS are otherwise healthy with normal renal function and coagulation profiles; the pathological process causing DSS involves a single organ, the vascular system, rather than affecting multiple organ systems as in many adult septic shock cases; and the time-period during which volume support is urgently required is short, 48–72 h, limiting the volume of colloid that is likely to be needed before the vasculopathy reverses and the child recovers (1).

Detailed evaluation of the efficacy and safety of the fluid regimens and crystalloid/colloid solutions in regular use for DSS resuscitation is certainly warranted but will require large and probably costly trials, especially if adequately powered to address the very real predicaments faced by clinicians who manage DSS on a daily basis—in particular how best to manage profound or recurrent shock. Similarly, while we must not underestimate the difficulties inherent in such research in children with life-threatening shock, trying to establish a more evidence-based approach to the use of blood products would be very helpful for clinicians faced with this difficult scenario. In the meanwhile, our current understanding of the principles determining intravascular volume regulation supports the use of isotonic crystalloids for primary resuscitation of DSS even in decompensated shock. Similarly, should colloids be deemed essential, management should be indivualized to ensure use of the smallest volume necessary to just maintain adequate circulation, ideally avoiding use of rapid boluses or large volumes of fluid.

## Summary and Conclusions

It is clear that, like all therapeutic interventions, parenteral fluids have both beneficial and adverse effects, and that these vary depending on the clinical context. Increasing efforts are being made to develop truly evidence-based guidelines for many critical care scenarios, yet such research studies rarely take place in the low-middle income countries where the disease burden is often considerable while facilities and resources are typically limited. As the geographical footprint of dengue expands, how best to manage DSS is likely to become an ever more pressing issue that will demand formal research efforts to address the crucial questions that remain. Until a safe and effective vaccine is widely deployed across dengue-endemic areas we must continually strive to make supportive care as good as it can be. But the evolution of the crystalloid/colloid debate over the years since Starling first proposed his model highlights the importance of continually re-evaluating the principles and practice of patient management (in this case fluid resuscitation) in the light of new knowledge; what we understand to be the best approach today may not remain so in the future.

## Author Contributions

DT: topic conceptualization for this review, literature review, writing – original draft preparation, writing – review and editing. HT and BW: literature review, writing – original draft preparation, writing – review and editing. All authors contributed to the article and approved the submitted version.

## Conflict of Interest

The authors declare that the research was conducted in the absence of any commercial or financial relationships that could be construed as a potential conflict of interest.
